# Pharmacists’ Role in Managing Patients with Chronic Lymphocytic Leukemia

**DOI:** 10.3390/pharmacy8020052

**Published:** 2020-03-27

**Authors:** Kevin Y. Chen, Kelly M. Brunk, Bianka A. Patel, Kurtis J. Stocker, Jessica J. Auten, Kaitlyn M. Buhlinger, Benyam Muluneh

**Affiliations:** 1 Department of Pharmacy, University of North Carolina Medical Center, Chapel Hill, NC 27514, USA; kevin.chen2@unchealth.unc.edu (K.Y.C.); Kelly.Brunk@unchealth.unc.edu (K.M.B.); bianka.patel@unchealth.unc.edu (B.A.P.); kurtis.stocker@unchealth.unc.edu (K.J.S.); Jessica.Auten@unchealth.unc.edu (J.J.A.); kaitlyn.buhlinger@unchealth.unc.edu (K.M.B.); 2 UNC Eshelman School of Pharmacy, University of North Carolina, Chapel Hill, NC 27514, USA

**Keywords:** CLL, pharmacist, ibrutinib, venetoclax, initiation, toxicity, monitoring, drug–drug interactions

## Abstract

Chronic lymphocytic leukemia (CLL) is a hematologic malignancy that has seen significant advances in care over the last 5 years with the approval of oral agents such as ibrutinib and venetoclax for the treatment of this disease. As such, there has been a substantial shift away from the traditional chemotherapy infusions which have allowed patients greater autonomy with oral cancer therapies. This paradigm shift poses new challenges for the medical team, including drug–drug interactions, adherence counseling, and financial toxicity. Pharmacists are uniquely trained and equipped to help to manage the changing landscape of CLL care. From identifying common medications which may impair ibrutinib clearance to ensuring patients are on the appropriate anti-infective prophylaxis while receiving obinutuzumab, pharmacists can play a vital role in ensuring the highest quality of patient care. Furthermore, additional credentialing of clinical pharmacists in select states allows for independent visits with the pharmacists, allowing for greater involvement, particularly for initiation of venetoclax and management of ibrutinib-induced toxicities. Pharmacists are essential to both expanding and enhancing the care of patients with CLL and should be leveraged to improve patient outcomes whenever possible.

## 1. Introduction

Chronic lymphocytic leukemia (CLL) is the most common leukemia in adults, with over 20,000 new cases each year in the United States [1]. CLL typically afflicts older adults, with a median age of diagnosis at 72 years. It is characterized by the proliferation and accumulation of mature B-cells within the blood, bone marrow, and lymphoid tissue and is a disease with a variable clinical course. Historically, the treatment of CLL primarily consisted of cytotoxic chemotherapy in combination with an anti-CD20 monoclonal antibody. In the past several years, important advances in the understanding of the pathogenesis of CLL have led to the development of novel therapies for CLL, resulting in a significant paradigm shift in the treatment approach [2].

Ibrutinib, a small-molecule Bruton tyrosine kinase (BTK) inhibitor, was the first orally-administered, targeted agent approved for CLL in 2014. Since then, another BTK inhibitor, acalabrutinib, two phosphatidylinositol-3-kinase (PI3K) inhibitors, idelalisib and duvelisib, and a B-cell lymphoma 2 (BCL-2) inhibitor, venetoclax, have also been approved for CLL [1]. Due to this growth in therapeutic options, choosing the optimal treatment for a patient with CLL has become a complex task that requires in-depth knowledge of drug therapy while taking into account patient characteristics such as age, cytogenetics, and comorbidities. The advent of these therapies has also brought with it a new set of practical considerations, including patient adherence, drug–drug interactions (DDIs), toxicity management, supportive care requirements, and medication access.

Pharmacists play a vital role in every aspect of care for patients with CLL. Through collaborative practice agreements, pharmacists can maximize their impact on a multidisciplinary team and take the lead in many of the processes required to guarantee patient success. Prior to the initiation of treatment, pharmacists identify DDIs and relevant drug administration concerns, which is especially important given that CLL patients are often older with several comorbidities and concurrent medications. Oral oncolytics are high-cost treatments, and financial barriers may limit their access to patients. Pharmacists are essential in overseeing medication access to the most appropriate treatment for patients by partnering with medication access specialists (usually technicians) and social workers [3]. Often, a strong partnership between clinical pharmacists and specialty pharmacists is necessary to create a closed-loop, patient-centered system. Having pharmacists involved operationally and clinically ensures patients are able to have timely access to and delivery of their drug as well as management of non-adherence and toxicities, which can lead to improved patient outcomes [4,5]. Pharmacists can also play a key role in educating patients and caregivers on the risks and benefits of treatment options, allowing for shared decision making and increased patient engagement [6].

Throughout treatment, pharmacists are well-positioned to address adherence to self-administered oral oncolytics. Pharmacists also ensure that patients receive appropriate infection prophylaxis and adequate monitoring for toxicities associated with each therapy. Each of the different therapeutic options, ranging from the anti-CD20 monoclonal antibodies to various oral targeted agents, have distinct recommendations for infection prophylaxis [7]. Tyrosine kinase inhibitors (TKIs), PI3K inhibitors, and venetoclax are all associated with a unique set of adverse effects and monitoring parameters [1]. The oncology care team often looks to the pharmacist for guidance when managing toxicities from chemotherapy, including dose modifications, treatment interruptions, supportive care, and even when to consider alternative therapies.

The aim of this review is to highlight the critical role of the pharmacist in the practical management of CLL and to emphasize specific areas in which pharmacists are most valuable.

## 2. Infection Prevention and Supportive Care

Pharmacists are uniquely positioned to manage and optimize both parenteral and oral therapies in patients with CLL. Parenteral therapies, including chemoimmunotherapy, as well as oral oncolytics have the potential for various adverse effects, often warranting myeloid growth factor support, infection prophylaxis, and antiemetics. In our practice, the pharmacist has a clinical visit with the patient before treatment initiation. At this visit, the pharmacist is responsible for the initiation of the appropriate prophylactic anti-infective and other supportive care agents to ensure optimal health outcomes.

Infectious complications during CLL therapy can generally be divided into early and late phases [7]. In the early period, which occurs in the first months of therapy, persistent neutropenia increases the risk of bacterial infections. This period is typically short-lived with most cytotoxic therapy but can be prolonged with fludarabine-based chemotherapy regimens. Growth factor support can be considered in patients with risk factors for prolonged neutropenia, such as renal or liver dysfunction. In the late period, which can last up to 2 years or more after therapy, T-cell dysfunction may occur, increasing the risk of both bacterial and opportunistic infections, including Listeria monocytogenes, Pneumocystis jiroveci pneumonia (PCP), cytomegalovirus (CMV), herpes simplex virus (HSV), and mycobacteria [7].

The necessity for prophylaxis for PCP, CMV, HSV, and hepatitis B virus (HBV) depends on the type of therapy used. Table 1 summarizes the antimicrobial prophylactic recommendations for specific drug therapies. For carriers of the hepatitis B virus (HBV), either chemotherapy or immunotherapy agents can increase the risk of HBV reactivation. Although the risk of HBV reactivation has been reported with oral ibrutinib and idelalisib, patients receiving anti-CD20 monoclonal antibody (mAb)-containing regimens, including rituximab, obinutuzumab, or ofatumumab, are at the highest risk [8]. Hepatitis B surface antigen (HBsAg) and core antibody (HBcAb) testing are recommended for all patients receiving anti-CD20 mAb-based regimens. In individuals who test positive for HBsAg and/or HBcAb, baseline quantitative PCR for HBV DNA should be obtained to determine viral load. HBV reactivation monitoring and prophylaxis with entecavir is recommended for high-risk patients receiving anti-CD20 mAb, alemtuzumab, and purine analogs. Lamivudine prophylaxis is not recommended due to the risk of resistance, which occurs in ~20% of patients [9]. Patients who receive entecavir prophylaxis have a 15% absolute risk reduction in HBV reactivation compared to those that do not receive prophylaxis (p = 0.027) [10]. Finally, recent administration of intravenous immunoglobulin (IVIG) can result in false positive serologies. Therefore, in these patients, it is important to consider repeat or PCR testing [11].

Beyond recommending the appropriate use of myeloid growth factor and infection prophylaxis, pharmacists play an important role in preventing and managing chemotherapy-induced nausea and vomiting (CINV). CINV occurs in up to 80% of patients on active therapy and remains a significant barrier to quality of life [12]. When uncontrolled, CINV can alter electrolytes and enteral nutrition, leading to detrimental effects on patient adherence and health outcomes [13]. The risk of emetogenicity varies depending on drug therapy. Most oral CLL chemotherapies, including acalabrutinib, ibrutinib, duvelisib, idelalisib, and venetoclax, have an emetogenic risk of <30% [14,15]. Immunotherapy with alemtuzumab and anti-CD20 monoclonal antibodies (mAbs) have a minimal emetogenic risk. Regimens with cytotoxic chemotherapy such as bendamustine and cyclophosphamide have a higher emetogenic risk. In our practice, pharmacists are often responsible for identifying drugs with emetogenic risk and subsequent management of nausea and vomiting.

## 3. Oral Chemotherapy Considerations

Oral chemotherapy provides a unique opportunity for patients to receive effective cancer therapies in the convenience of their own home, while also providing pharmacists with the chance to expand services that address unmet needs on the healthcare team. Over the past 5 years, small molecule inhibitors have changed the treatment approach to CLL and underscore the need and opportunities for pharmacist involvement in patient care. By providing services like comprehensive medication management, patient education, and medication access coordination, pharmacists improve health outcomes and patient satisfaction [4,16].

Comprehensive medication review not only encompasses a detailed medication reconciliation but also allows pharmacists to identify medication-related problems and access issues. One study quantified the impact of pharmacy student-driven medication reconciliation and found that 88% of patients had ≥1 discrepancy on their medication list and 11.4% of patients had a medication-related problem [17]. While it is difficult to quantify patient outcomes as a direct result of medication reconciliation, DDIs identified in these encounters can have serious treatment implications. Ibrutinib, venetoclax, and acalabrutinib are small molecule oral chemotherapy agents that are included in the guidelines as preferred first-line treatments for CLL. Each of these agents are metabolized by the cytochrome P450 (CYP) 3A enzyme and thus require dose adjustments for concomitant administration with 3A inducers and inhibitors [18,19,20]. Administration of these agents with interacting medications can result in excessive toxicities or loss of efficacy, both of which negatively impact patient outcomes. DDIs become particularly relevant in CLL, as these patients are often diagnosed in the later part of their lives and are already managing many other comorbid conditions. Additionally, individual agents have specific administration instructions, such as separating acalabrutinb and acid suppressing agents and taking venetoclax with food to optimize medication absorption, which complicates appropriate medication adherence in patients with a multitude of other medications [18,20]. This further reinforces the importance of pharmacist support for CLL patients on oral therapies.

Patient education and adherence is an integral part of successful oral chemotherapy treatment and is often a collaborative effort among patients, family members, providers, and pharmacists. Successful patient counseling includes the indication, dosing regimen, supportive care, adverse effects, storage, and administration, as well as medication reconciliation [21]. One observational study quantified patient satisfaction after an initial pharmacy-led education encounter prior to starting chemotherapy and found that 86% of patients felt that meeting with a pharmacist was “absolutely necessary.” Furthermore, 83% of patients stated that they would be willing to pay for this pharmacy-driven service [4]. While there is a clear benefit to initial chemotherapy education, adherence and continued follow-up remain significant barriers to optimal health outcomes [22].

One study of individuals with chronic myeloid leukemia (CML) found that patients with less than 90% adherence to imatinib only had a 28% chance of achieving major molecular response, as compared to a 94% chance in patients with adherence greater than 90% [23]. When a pharmacist is involved in follow-up and adherence management for oral TKIs in CML, adherence improves from 66% to 88% [24]. Similarly, a single-center retrospective analysis of CLL and non-Hodgkin’s lymphoma patients on ibrutinib demonstrated that poor medication adherence (<80%) was associated with worse progression-free survival (PFS) in the subset of CLL patients [25]. In fact, missing just eight consecutive doses of ibrutinib is an important predictor of PFS and further highlights the need for close follow-up and continuity of care [26]. Ensuring appropriate adherence to oral therapies in CLL is a great opportunity for pharmacists to improve clinical outcomes; however, financial toxicity affects both patients and their families and remains a significant barrier to care.

**Figure 1:**
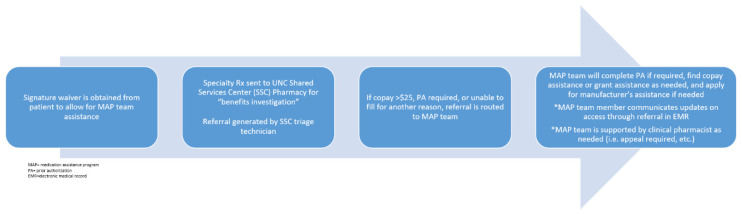
Medication assistance program**.**

As the medication therapy experts, pharmacists have traditionally played a key role in the acquisition of high cost medications; however, the incorporation of embedded medication assistance program (MAP) pharmacy technicians at our institution has significantly improved medication access. By assisting low-income and uninsured patients in need of high-cost medications, embedded MAP technicians work with clinical pharmacists to minimize treatment delays. This team-based approach to medication access makes sure that prior authorizations, manufacturer assistance, and grant applications are navigated quickly and efficiently in order to minimize financial toxicity and out-of-pocket costs. In order to quantify the financial impact of CLL treatments, Chen et al. developed a model to project the cost of CLL management after the incorporation of small molecule inhibitors into clinical practice [27]. The authors estimated that the annual cost of CLL management in 2011, $0.74 billion, would rise to $5.13 billion by 2025. Furthermore, they predicted that out-of-pocket costs would increase from $9200 per patient in 2011 to $57,000 per patient by 2025 [27]. These are unsurprising results given the current lack of defined therapy duration with many oral targeted therapies, and the improved patient response seen with these treatments. The high costs associated with these medications not only place undue stress on patients but also present many logistical challenges for the team during treatment initiation. Our institution-specific medication access which incorporates pharmacists and pharmacy technicians is outlined below (Figure 1). Pharmacists are vital in helping to navigate these obstacles and guarantee patients receive affordable and timely access to treatment.

## 4. Managing Toxicities of B-Cell Signaling Inhibitors

Common venetoclax toxicities include myelosuppression, increased infection risk, and development of tumor lysis syndrome (TLS). Management of these toxicities includes dose interruptions and reductions, neutrophil growth factors, and supportive care TLS medications such as allopurinol, phosphate binders, and intravenous (IV) fluids. Pharmacists can play a role in managing each of these toxicities. In contrast, Bruton kinase inhibitors (ibrutinib and acalabrutinib) have additional adverse effects, including increased rates of hypertension, bleeding, and development of new atrial and ventricular arrhythmias [28,29,30]. Pharmacists can help with educating the medical team and the patient about the importance of monitoring for these toxicities in order to make early referrals to a cardiologist.

Ibrutinib-induced hypertension was reported in only a modest amount of patients during clinical trials; however, emerging data suggest that this rate may be significantly higher than initially reported [31]. A recent single-centered retrospective review examined the incidence of new or worsened hypertension (defined as systolic blood pressure (SBP) >130 mm Hg) in 562 patients who received ibrutinib [30]. The authors found that 78.3% experienced new or worsened hypertension, with 17.7% of those experiencing grade 3 hypertension (SBP >160/100 mm Hg). Furthermore, they found a correlation between the incidence of major adverse cardiac events (MACEs) and new or worsening hypertension. While these adverse effects can be mitigated with antihypertensive therapies, it is important to note that no specific anti-hypertensive agent was found to be superior in managing ibrutinib-induced hypertension. Therefore, pharmacists can play a vital role in selecting the most appropriate anti-hypertensive agent for managing ibrutinib-induced hypertension while considering underlying comorbidities such as heart failure, diabetes, or renal artery stenosis. For example, beta-blockers, angiotensin-converting enzyme (ACE)-inhibitors or angiotensin II receptor blockers (ARBs), and calcium channel blockers can be preferentially used in patients with prior myocardial infarction, diabetes, and angina, respectively. Conversely, it is prudent to avoid certain agents in high-risk populations, such as ACE-inhibitors or ARBs with renal artery stenosis or calcium channel blockers in decompensated heart failure. Because of their medication-related expertise, pharmacists are able to choose the optimal anti-hypertensive agent to manage both ibrutinib-induced hypertension and other comorbidities.

Ibrutinib also increases the risk for bleeding, including both minor bleeding, such as bruising and epistaxis, as well as life-threatening major bleeds. Depending on the study, the rates of bleeding range from 7% to 56% for all grades or 1% to 19% for grade 3 and above [29,32]. As there are currently no therapies for the treatment of ibrutinib-induced bleeding, the recommended management strategies aim to prevent and reduce the risk of bleeding [33]. One retrospective analysis conducted in 70 patients identified several predisposing risk factors for increased bleeding [32]. These risk factors included international normalized ratio (INR) >1.5, hemoglobin < 12 mg/dL, use of interacting medications, and use of antiplatelet and/or anticoagulation agents. In high-risk patients, pharmacists are well suited to facilitate multidisciplinary discussions between a patient’s cardiologist and oncologist on the risks and benefits of continuing antiplatelet or anticoagulation therapy in patients with cardiac stents or venous thromboembolisms, respectively. Pharmacists can also play an important role in the identification of medications that may interact with ibrutinib or various anticoagulation and antiplatelet agents through a comprehensive medication review process. In addition, they are able to identify over-the-counter medications, vitamins, and supplements that may increase the risk of bleeding. These agents can range from nonsteroidal anti-inflammatory drugs and high-dose steroids to fish oils and vitamin E. Pharmacists can provide vital knowledge on how to prevent drug–drug and drug–disease interactions in these patients.

Although incidence of ibrutinib-induced atrial fibrillation is low (~6%), it poses a unique challenge of balancing bleeding and thrombosis risk, since systemic anticoagulation is a risk factor for ibrutinib-induced bleeding [32,34]. Additionally, given the median age of CLL diagnosis, most patients who develop atrial fibrillation would benefit from anticoagulation for stroke prevention. In the absence of clinical evidence supporting the use of a specific anticoagulant, emphasis must be placed on identifying patients who are at higher risk for developing atrial fibrillation and bleeding. Patients with pre-existing hypertension, heart failure, diabetes, obesity, valvular heart disease, and chronic obstructive pulmonary disease may have an increased risk for developing atrial fibrillation, while patients with underlying coagulation disorders or anemia may be at greater risk for bleeding [35]. Firstly, pharmacists may assist oncologists with making initial treatment decisions for CLL based on patient risk factors and identifying patients who are better candidates for alternative CLL therapies, such as venetoclax, based off their comorbidities or other indicated concomitant medications. Secondly, pharmacists are able to provide comprehensive medication reviews, which help to prevent any potential DDIs with common rate and rhythm control agents, including diltiazem, verapamil, and amiodarone. Lastly, pharmacists in states with collaborative practice agreements may also assist with closer clinical monitoring by conducting independent follow-up visits with high-risk patients and order labs and medications where appropriate.

## 5. A Pharmacist-Led Program for Venetoclax Ramp-Up

As described thus far, CLL treatment has become increasingly complex and provides incredible opportunities for pharmacist intervention. In a clinic setting that allows for pharmacists to be intimately involved in patient care, pharmacists can develop programs around management of these complex regimens. One particular scenario in which pharmacists are particularly invaluable is in initiation of venetoclax for CLL, and here, we report a program designed at our institution for pharmacists leading in initiation and monitoring of venetoclax.

**Figure 2:**
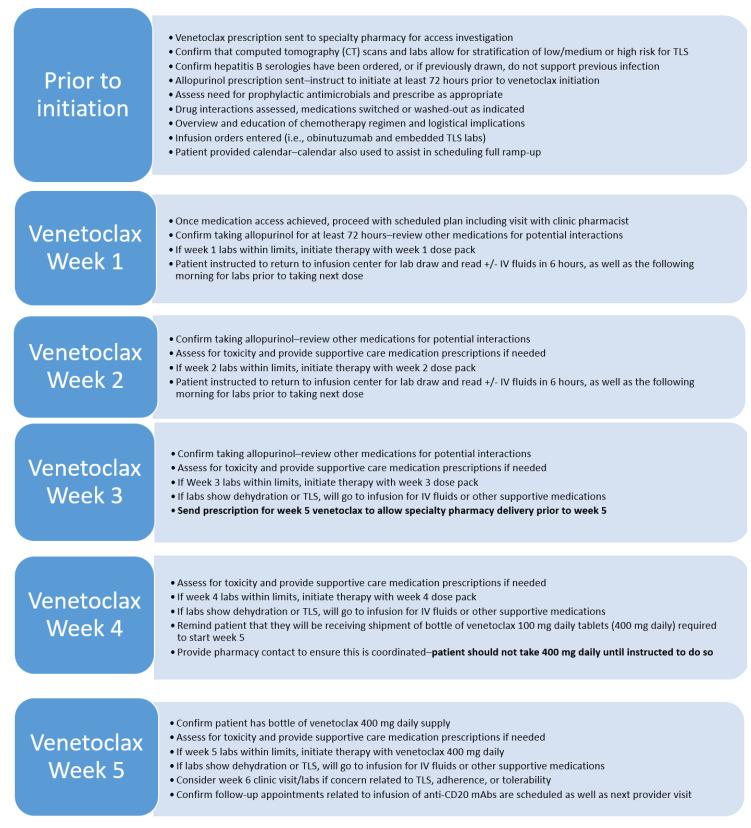
Pharmacist-led venetoclax initiation program.

When venetoclax was first studied in the phase 1 setting in relapsed/refractory patients, significant clinical sequelae were noted in several patients, including a death associated with TLS [36]. When venetoclax was studied thereafter, a dose-escalation period with close monitoring was utilized alongside prophylactic allopurinol, resulting in successful reduction of adverse events associated with TLS [37]. Venetoclax prescribing information outlines a five-week ramp-up period for CLL, with a specific set of requirements for the safe initiation of venetoclax [18]. These recommendations are further complicated when venetoclax is used in combination with an anti-CD20 mAb. The need for such intricate monitoring and careful design of a treatment plan is what sparked the need for a pharmacist-led venetoclax ramp-up program. Some important aspects of this program are highlighted below (Figure 2).

### 5.1. Treatment Initiation

At our institution, any patient being considered for initiation of a venetoclax-containing regimen will immediately be seen by the clinical pharmacist. The first step in the process is to determine appropriate TLS prophylaxis (Table S1). Along with a complete blood count (CBC) with differential and comprehensive metabolic panel (CMP), cross-sectional imaging should be scheduled as soon as possible in order to assess lymph node size. A prescription for allopurinol 300 mg daily must be sent to the patient’s pharmacy with instructions to start this medication at least 3 days prior to treatment initiation. After TLS prophylaxis, the next step is to establish the patient’s anti-infective regimen (Table 1).

Prior to treatment initiation of venetoclax at our institution, a venetoclax prescription is sent to our institutional specialty pharmacy, which notifies a group of embedded MAP technicians to begin the process of access investigation. A member of the pharmacy team then instructs the dispensing specialty pharmacy to send the venetoclax starter pack directly to our institution’s cancer hospital infusion pharmacy in order to allow efficient facilitation of the weekly ramp-up by the clinical pharmacist. The clinical pharmacist will see the patient to provide education prior to treatment initiation. Counseling the patient on the venetoclax-containing regimen should include a focus on dosing, administration, adherence, and the medication access process, as well as an overview of adverse effects. At this visit, a comprehensive medication review and identification of interacting medications is essential. If a patient is on concomitant interacting medications, the pharmacist is responsible for developing a plan to wash out/switch medications as necessary.

Patients must also be educated on TLS and the critical importance of adhering to allopurinol and adequate hydration. Patients should be advised on the logistics of TLS monitoring during the ramp-up phase. After TLS risk has been assessed, the pharmacist will create a calendar to assist in scheduling and to ensure patient understanding of monitoring requirements (Figure S1). If a patient is considered high-risk, the pharmacist and provider work with the oncology nurse navigator to schedule a 48-hour admission during week 1 and week 2 dose ramp-ups. In this case, the inpatient clinical pharmacist will facilitate retrieving the venetoclax supply from the cancer hospital pharmacy for inpatient use.

### 5.2. Scheduling and Ramp-Up Monitoring

The clinical pharmacist is the liaison for logistical needs during the dose-escalation period and works closely with the schedulers and nurse navigators to plan the entire ramp-up (Figure 2). The patient calendar is adjusted accordingly for recommended scheduling based on CLL regimen/TLS risk (Table S2). With or without a provider appointment, weekly appointments with the clinical pharmacist are necessary to ensure that the patient receives the correct venetoclax dose pack. On the same days of these appointments, the patient has a scheduled visit at the infusion center, where IV fluids or other supportive transfusions can be administered if needed. For nursing and patient convenience, laboratory draws should occur as early in the day as possible.

During the ramp-up phase, the clinical pharmacist evaluates the patient’s weekly CBC and CMP. If these indicate that the patient is dehydrated, they will receive IV fluids and will be re-educated on the importance of drinking 6 to 8 glasses of water daily. If laboratory values indicate concern for TLS, the pharmacist will manage the patient as clinically necessary (e.g., IV fluids). Once the laboratory values are within normal ranges, the pharmacist will provide the subsequent dose pack to the patient. In addition to these steps, the clinical pharmacist will conduct a comprehensive assessment during each ramp-up appointment. During these times, the pharmacist will evaluate adverse effects, identify supportive care needs, review and update the patient’s medication list, and re-emphasize important drug and disease counseling.

Since the pharmacist is acutely aware of how well the patient is tolerating each ramp-up dose, they are ideally positioned to coordinate delivery of the final ramp-up dose. Typically, during week 3 of the ramp-up, the pharmacist coordinates the home delivery of the week 5 ramp-up dose. This planning ensures that when the patient is ready to start venetoclax 400 mg, the prescription will be in the patient’s possession.

## 6. Conclusions

Pharmacists play an integral role in the comprehensive management of patients with CLL. Their unique perspective and medication-related expertise add value throughout the continuum of care. From providing chemotherapy education and comprehensive medication management to conducting independent clinical follow-up visits, pharmacists are well-positioned to take the lead in many aspects of care for these patients. Through collaborative practice agreements and by working alongside physicians, advanced practice providers, nurses, and other support staff, pharmacists are able to practice at an elevated level and utilize the full extent of their training. CLL is a malignancy in which pharmacist collaboration is imperative given the numerous toxicities, DDIs, and logistics of initiating oral agents. Many of the processes in which pharmacists have the greatest degree of involvement at our institution are highlighted here and may be adapted to other institutions and practice settings.

## Figures and Tables

**Table 1:** Infection prophylaxis with chronic lymphocytic leukemia (CLL) therapies.

Agent	HSV and Varicella *	CMV	HBV	PCP
Acalabrutinib	No	No	No	No
Duvelisib	No	Consider	No	Yes **
Ibrutinib	No	No	No	Consider ***
Idelalisib	No	Consider	No	Yes
Venetoclax	No	No	No	No
Purines, Bendamustine	Yes	Consider	No	Yes
Anti-CD20	No	No	Yes (HBsAg+ or HBcAb+)	No
Alemtuzumab	Yes	Yes (weekly CMV PCR)	No	Yes

* Consider valacyclovir 500 mg daily for high-risk patients (e.g., elderly or recent history of herpes or shingles reactivation). ** Pneumocystis jiroveci pneumonia (PCP) prophylaxis with sulfamethoxazole/trimethoprim or equivalent during treatment and until the absolute CD4+ T-cell count is >200 cells/uL. *** PCP prophylaxis may not be warranted given the low risk of PCP.

## Supplementary Materials

The following are available online at https://www.mdpi.com/2226-4787/8/2/52/s1, Figure S1: Venetoclax initiation calendar, Table S1: Recommendations for TLS prophylaxis for CLL patients (per package insert), Table S2: Scheduling venetoclax and obinutuzumab ramp-up in CLL.

## References

[1] Hallek M. (2019). Chronic lymphocytic leukemia: 2020 update on diagnosis, risk stratification and treatment. Am. J. Hematol..

[2] Hallek M. (2017). Chronic lymphocytic leukemia: 2017 update on diagnosis, risk stratification and treatment. Am. J. Hematol..

[3] Mitchell A., Muluneh B., Patel R., Basch E. (2018). Pharmaceutical assistance programs for cancer patients in the era of orally administered chemotherapeutics. J. Oncol. Pharm. Pract..

[4] Mackler E., Segal E.M., Muluneh B., Jeffers K., Carmichael J. (2019). Hematology/Oncology Pharmacist Association Best Practices for the Management of Oral Oncolytic Therapy: Pharmacy Practice Standard. J. Oncol. Pract..

[5] Muluneh B., Schneider M., Faso A., Amerine L., Daniels R., Crisp B., Valgus J., Savage S. (2018). Improved Adherence Rates and Clinical Outcomes of an Integrated, Closed-Loop, Pharmacist-Led Oral Chemotherapy Management Program. J. Oncol. Pharm. Pract..

[6] Rocque G.B., Williams C.P., Halilova K.I., Borate U., Jackson B.E., Van Laar E.S., Pisu M., Butler T.W., Davis R.S., Mehta A. (2018). Improving shared decision-making in chronic lymphocytic leukemia through multidisciplinary education. Transl. Behav. Med..

[7] Tadmor T., Welsau M., Hus I. (2018). A review of the infection pathogenesis and prophylaxis recommendations in patients with chronic lymphocytic leukemia. Expert Rev. Hematol..

[8] Kusumoto S., Arcaini L., Hong X., Jin J., Kim W.S., Kwong Y.L., Peters M.G., Tanaka Y., Zelenetz A.D., Kuriki H. (2019). Risk of HBV reactivation in patients with B-cell lymphomas receiving obinutuzumab or rituximab immunochemotherapy. Blood.

[9] Kim S.J., Jsu C., Song Y.Q., Tay K., Hong X.N., Cao J., Kim J.S., Eom H.S., Lee J.H., Zhu J. (2013). Hepatitis B virus reactivation in B-cell lymphoma patients treated with rituximab: Analysis from the Asia Lymphoma Study Group. Eur. J. Cancer.

[10] Huang Y.H., Hsiao L.T., Hong Y.C., Chiou T.J., Yu Y.B., Gau J.P., Liu C.Y., Yang M.H., Tzeng C.H., Lee P.C. (2013). Randomized controlled trial of entecavir prophylaxis for rituximab-associated hepatitis B virus reactivation in patients with lymphoma and resolved hepatitis B. J. Clin. Oncol..

[11] Arnold D.M., Crowther M.A., Meyer R.M., Carruthers J., Ditomasso J., Heddle N.M., McLeod A., Kelton J.G. (2010). Misleading hepatitis B test results due to intravenous immunoglobulin administration: Implications for a clinical trial of rituximab in immune thrombocytopenia. Transfusion.

[12] Mustian K.M., Devine K., Ryan J.L., Janelsins M.C., Sprod L.K., Peppone L.J., Candelario G.D., Mohile S.G., Morrow G.R. (2011). Treatment of Nausea and Vomiting During Chemotherapy. US Oncol. Hematol..

[13] Hamadani M., Chaudhary L., Awan F.T., Khan J.K., Kojouri K., Ozer H., Tfayli A. (2007). Management of platinum-based chemotherapy-induced acute nausea and vomiting: Is there a superior serotonin receptor antagonist?. J. Oncol. Pharm. Pract..

[14] Hesketh P.J., Kris M.G., Basch E., Bohlke K., Barbour S.Y., Clark-Snow R.A., Danso M.A., Dennis K., Dupuis L.L., Dusetzina S.B. (2017). Antiemetics: American Society of Clinical Oncology Clinical Practice Guideline Update. J. Clin. Oncol..

[15] National Comprehensive Cancer Network Antiemesis (Version 1.2020). https://www.nccn.org/professionals/physician_gls/pdf/antiemesis.pdf.

[16] McKee M., Frei B.L., Garcia A., Fike D., Soefje S.A. (2011). Impact of clinical pharmacy services on patients in an outpatient chemotherapy academic clinic. J. Oncol. Pharm. Pract..

[17] Ashjian E., Salamin L.B., Eschenburg K., Kraft S., Mackler E. (2015). Evaluation of outpatient medication reconciliation involving student pharmacists at a comprehensive cancer center. J. Am. Pharm. Assoc..

[18] (2019). Venclexta (Venetoclax) [Prescribing Information].

[19] (2019). Imbruvica (Ibrutinib) [Prescribing Information].

[20] (2019). Calquence (Acalabrutinib) [Prescribing Information].

[21] Felton M.A., van Londen G.J., Marcum Z.A. (2016). Medication adherence to oral cancer therapy: The promising role of the pharmacist. J. Oncol. Pharm. Pract..

[22] Battis B., Clifford L., Huq M., Pejoro E., Mambourg S. (2017). The impacts of a pharmacist-managed outpatient clinic and chemotherapy-directed electronic order sets for monitoring oral chemotherapy. J. Oncol. Pharm. Pract..

[23] Marin D., Bazeos A., Mahon F.X., Eliasson L., Milojkovic D. (2010). Adherence is the critical factor for achieving molecular responses in patients with chronic myeloid leukemia who achieve complete cytogenetic responses on imatinib. J. Clin. Oncol..

[24] Lam M.S., Cheung N. (2016). Impact of oncology pharmacist-managed oral anticancer therapy in patients with chronic myelogenous leukemia. J. Oncol. Pharm. Pract..

[25] Williams A., Baran A., Casulo C., Reagan P., Friedberg J.W., Helber M., Moore J., Baloga E., Zent C.S., Barr P.M. (2019). Ibrutinib dose adherence and therapeutic efficacy in non-hodgkin lymphoma: A single-center experience. Clin. Lymphoma Myeloma Leuk..

[26] Barr P.M., Brown J.R., Hillmen P., O’Brien S., Barrientos J.C., Reddy N.M., Coutre S., Mulligan S.P., Jaeger U., Furman R.R. (2017). Impact of ibrutinib dose adherence on therapeutic efficacy in patients with previously treated CLL/SLL. Blood.

[27] Chen Q., Jain N., Ayer T., Wierda W.G., Flowers C.R., O’Brien S.M., Keating M.J., Kantarjian H.M., Chhatwal J. (2017). Economic Burden of Chronic Lymphocytic Leukemia in the Era of Oral Targeted Therapies in the United States. J. Clin. Oncol..

[28] Salem J.E., Manouchehri A., Bretagne M., Lebrun-Vignes B., Groarke J.D., Johnson D.B., Yang T., Reddy N.M., Funck-Brentano C., Brown J.R. (2019). Cardiovascular toxicities associated with ibrutinib. J. Am. Coll. Cardiol..

[29] Stephens D.M., Byrd J.C. (2019). How I manage ibrutinib intolerance and complications in patients with chronic lymphocytic leukemia. Blood.

[30] Dickerson T., Wiczer T., Waller A., Philippon J., Porter K., Haddad D., Guha A., Rogers K.A., Bhat S., Byrd J.C. (2019). Hypertension and incident cardiovascular events following ibrutinib initiation. Blood.

[31] O’Brien S., Hillmen P., Coutre S., Barr P.M., Fraser G., Tedeschi A., Burger J.A., Dilhuydy M.S., Hess G., Moreno C. (2018). Safety analysis of four randomized controlled studies of ibrutinib in patients with chronic lymphocytic leukemia/small lymphocytic lymphoma or mantle cell lymphoma. Clin. Lymphoma Myeloma Leuk..

[32] Mock J., Kunk P.R., Palkimas S., Sen J.M., Devitt M., Horton B., Portell C.A., Williams M.E., Maitland H. (2018). Risk of major bleeding with ibrutinib. Clin. Lymphoma Myeloma Leuk..

[33] Shatzel J.J., Olson S.R., Tao D.L., McCarty O.J., Danilov A.V., DeLoughery T.G. (2017). Ibrutinib-associated bleeding: Pathogenesis, management and risk reduction strategies. J. Thromb. Haemost..

[34] Boriani G., Corradini P., Cuneo A., Falanga A., Foa R., Gaidano G., Ghia P.P., Martelli M., Marasca R., Massaia M. (2018). Practical management of ibrutinib in the real life: Focus on atrial fibrillation and bleeding. Hematol. Oncol..

[35] Thorp B.C., Badoux X. (2018). Atrial fibrillation as a complication of ibrutinib therapy: Clinical features and challenges of management. Leuk. Lymphoma.

[36] Roberts A.W., Davids M.S., Pagel J.M., Kahl B.S., Puvvada S.D., Gerecitano J.F., Kipps T.J., Anderson M.A., Brown J.R., Gressick L. (2016). Targeting BCL2 with venetoclax in relapsed chronic lymphocytic leukemia. N. Engl. J. Med..

[37] Stilgenbauer S., Eichhorst B., Schetelig J., Coutre S., Seymour J.F., Munir T., Puvvada S.D., Wendtner C.M., Roberts A.W., Jurczak W. (2016). Venetoclax in relapsed or refractory chronic lymphocytic leukaemia with 17p deletion: A multicentre, open-label, phase 2 study. Lancet Oncol..

