# Supplementary material for: Pharmacists’ Role in Managing Patients with Chronic Lymphocytic Leukemia
Source: Pharmacy (Basel). 2020 Mar 27;8(2):52. doi: 10.3390/pharmacy8020052 (PMC7355755; doi:10.3390/pharmacy8020052)
Supplement: Supplementary file 1 [file pharmacy-08-00052-s001.pdf]

Supplementary Figure 1: Venetoclax Initiation Calendar.

◂ September | October 2019 | November ▸

Sun	Mon	Tue	Wed	Thu	Fri	Sat
6	7 Day 1<br>Labs<br>Clinic Visit<br>Obinutuzumab IV	8<br>Labs<br>Obinutuzumab IV	9	10	11	12
13	14 Day 8	15<br>Labs<br>Obinutuzumab IV	16	17	18	19
20	21 Day 15	22<br>Labs<br>Obinutuzumab IV	23	24	25	26
27	28 Day 22	29<br>Labs<br>Clinic<br>Venetoclax 20 mg<br>(infusion for 6-8hr lab monitoring)	30<br>Labs<br>Infusion (lab follow-up)<br>Venetoclax 20 mg	31<br>Venetoclax 20 mg		

◀ October | November 2019 | December ▶

Sun	Mon	Tue	Wed	Thu	Fri	Sat
					1	2

					Venetoclax 20 mg	Venetoclax 20 mg
3<br>Venetoclax 20 mg	4 Day 1<br>Venetoclax 20 mg	5<br>Labs<br>Clinic<br>Venetoclax 50 mg<br>Infusion – Obinutuzumab IV PLUS<br>infusion for 6-8hr lab monitoring	6<br>Labs<br>Infusion (lab follow-up)<br>Venetoclax 50 mg	7<br>Venetoclax 50 mg	8<br>Venetoclax 50 mg	9<br>Venetoclax 50 mg
10<br>Venetoclax 50 mg	11 Day 8<br>Venetoclax 50 mg	12<br>Labs<br>Clinic<br>Venetoclax 100 mg	13<br>Venetoclax 100 mg	14<br>Venetoclax 100 mg	15<br>Venetoclax 100 mg	16<br>Venetoclax 100 mg
17<br>Venetoclax 100 mg	18 Day 15<br>Venetoclax 100 mg	19<br>Labs<br>Clinic<br>Venetoclax 200 mg	20<br>Venetoclax 200 mg	21<br>Venetoclax 200 mg	22<br>Venetoclax 200 mg	23<br>Venetoclax 200 mg
24<br>Venetoclax 200 mg	25 Day 22<br>Venetoclax 200 mg	26<br>Labs<br>Clinic<br>Venetoclax 400 mg	27<br>Venetoclax 400 mg	28<br>Venetoclax 400 mg	29<br>Venetoclax 400 mg	30<br>Venetoclax 400 mg

◂ November — December 2019 — January ▸

Sun	Mon	Tue	Wed	Thu	Fri	Sat

1<br><br>Venetoclax 400 mg	2<br><br>Venetoclax 400 mg	3 Day 1<br>Labs<br>Clinic<br>Obinutuzumab IV<br>Venetoclax 400 mg	4<br><br>Venetoclax 400 mg	5<br><br>Venetoclax 400 mg	6<br><br>Venetoclax 400 mg	7<br><br>Venetoclax 400 mg

Supplementary Table 1: Recommendations for TLS prophylaxis for CLL patients (per package insert).

Tumor Burden		Prophylaxis		Blood Chemistry Monitoring
		Hydration	Anti-uricemic	Setting and frequency of assessments
Low	All LN < 5 cm<br><br>AND<br><br>ALC < 25 x 10^9/L	Oral (1.5-2L)	Allopurinol	Outpatient:<br>For first dose of 20 mg and 50 mg: Pre-dose, 6 to 8 hours, 24 hours<br><br>For subsequent ramp-up doses: Pre-dose
Medium	Any LN 5 – 10 cm<br><br>OR<br><br>ALC ≥ 25 x 10^9/L	Oral (1.5-2L) and consider additional IV	Allopurinol	Outpatient:<br>For first dose of 20 mg and 50 mg: Pre-dose, 6 to 8 hours, 24 hours<br><br>For subsequent ramp-up doses: Pre-dose<br><br>For first dose of 20 mg and 50 mg: Consider hospitalization for patients with CrCl < 80 ml/min; see below for monitoring in hospital

High	Any LN ≥ 10 cm<br><br>OR<br><br>ALC ≥ 25 x 10^9/L<br><br>AND<br><br>Any LN ≥ 5 cm	Oral (1.5-2L)<br>AND<br>IV (150-200 mL/h as tolerated)	Allopurinol and consider rasburicase if baseline uric acid is elevated	In hospital:<br>For first dose of 20 mg and 50 mg:<br>Pre-dose, 4, 8, 12, and 24 hours<br><br>Outpatient:<br>For subsequent ramp-up doses: Pre-dose, 6 to 8 hours, 24 hours

Supplementary Table 2: Scheduling Venetoclax and Obinutuzumab Ramp-Up in CLL.

Low/Medium Risk CLL Patients	High Risk CLL Patients
Cycle 1 Day 1, 2, 8, 15:<br>· Labs (CMP, CBC, etc.)<br>· Infusion (obinutuzumab)<br><br>Ven Week 1 Day 1 (Cycle 1 Day 22)<br>· Labs 0800<br>· Clinic 0900<br>o PharmD<br>o Provider<br>· Infusion 1600<br><br>Week 1 Day 2<br>· Labs 0800<br>· Infusion 0830<br><br>Week 2 Day 1 (Cycle 2 Day 1)<br>· Labs 0800<br>· Clinic 0900<br>o PharmD<br>o Provider<br>· Infusion 1200 (obinutuzumab)<br>o 6 hour post ven labs<br><br>Week 2 Day 2<br>· Labs 0800<br>· Infusion 0830<br><br>Week 3 – 5 Day 1<br>· Labs 0800<br>· Clinic 0900<br>o PharmD<br>o Provider<br>· Infusion 1000 – possible fluids<br><br>Cycle 3-6 Day 1	Cycle 1 Day 1, 2, 8, 15:<br>· Labs (CMP, CBC, etc.)<br>· Infusion (obinutuzumab)<br>_<br>Ven Week 1 Day 1 (Cycle 1 Day 22)<br>· Admission required<br><br>Ven Week 1 Day 6 (day prior to admission)<br>· Labs<br>· PharmD or Provider<br>· Infusion (Cycle 2 obinutuzumab)<br><br>Week 2 Day 1<br>· Admission required<br><br>Week 3 – 5 Day 1<br>· Labs 0800<br>· Clinic 0900<br>o PharmD<br>o Provider<br>· Infusion 1600<br><br>Week 3 – 5 Day 2<br>· Labs 0800<br>· Infusion 0830<br><br>Cycle 3-6 Day 1<br>· Labs<br>· Provider<br>· Infusion (obinutuzumab)

· Labs<br>· Provider<br>· Infusion (obinutuzumab)	
